# Supplementary material for: The causal role of circulating inflammatory markers in osteoporosis: a bidirectional Mendelian randomized study
Source: Front Immunol. 2024 Jul 18;15:1412298. doi: 10.3389/fimmu.2024.1412298 (PMC11291241; doi:10.3389/fimmu.2024.1412298)
Supplement: Supplementary file 1 [file DataSheet_1.zip › supplementary material/OSTEOPOROSIS-CIM/GCST90274803.tsv.gz_leave_one_out.pdf]

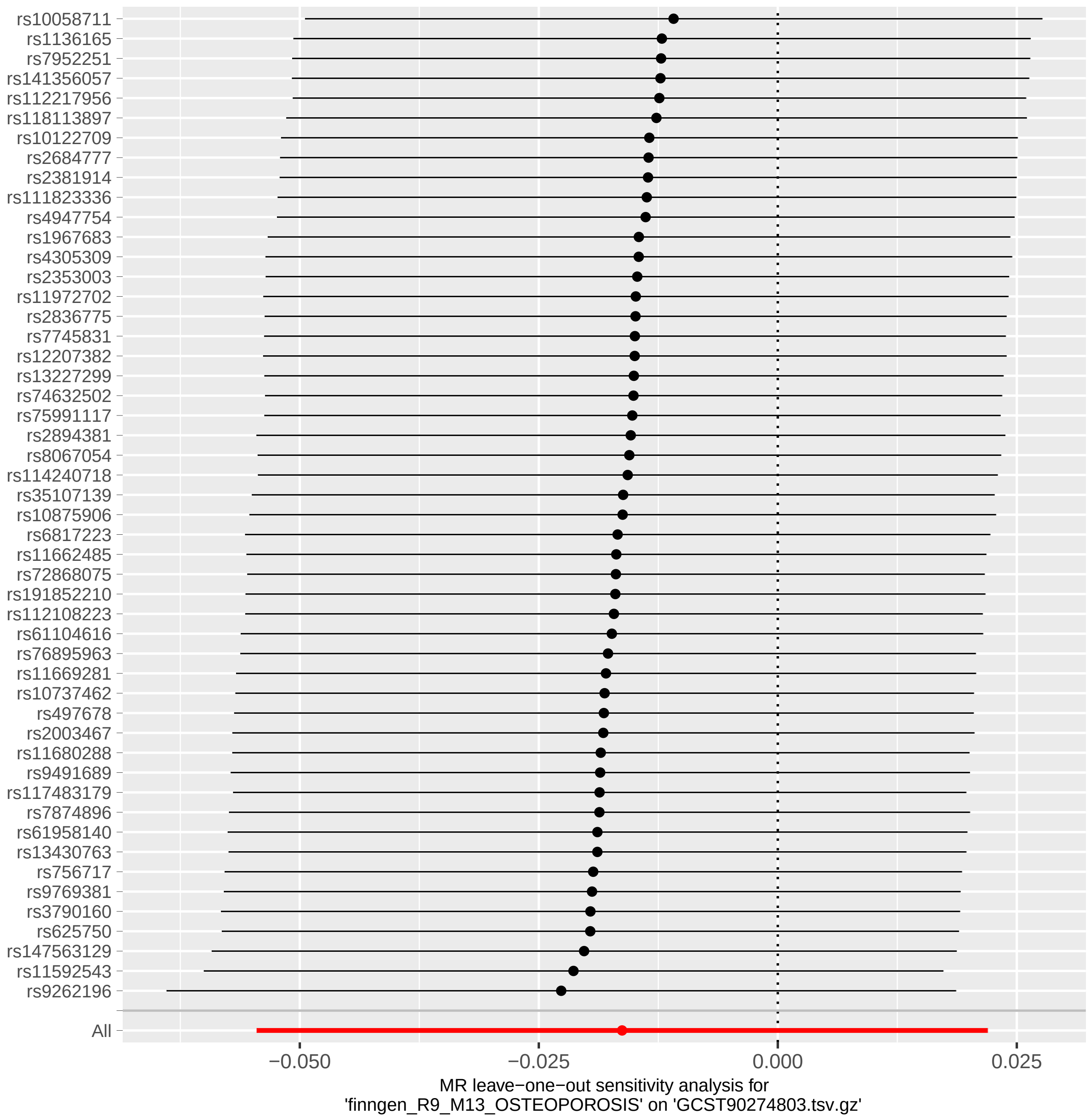
rs10058711
rs1136165
rs7952251
rs141356057
rs112217956
rs118113897
rs10122709
rs2684777
rs2381914
rs111823336
rs4947754
rs1967683
rs4305309
rs2353003
rs11972702
rs2836775
rs7745831
rs12207382
rs13227299
rs74632502
rs75991117
rs2894381
rs8067054
rs114240718
rs35107139
rs10875906
rs6817223
rs11662485
rs72868075
rs191852210
rs112108223
rs61104616
rs76895963
rs11669281
rs10737462
rs497678
rs2003467
rs11680288
rs9491689
rs117483179
rs7874896
rs61958140
rs13430763
rs756717
rs9769381
rs3790160
rs625750
rs147563129
rs11592543
rs9262196
All
−0.050
−0.025
0.000
0.025
MR leave−one−out sensitivity analysis for
'finngen_R9_M13_OSTEOPOROSIS' on 'GCST90274803.tsv.gz'
